# Natural Fiber Reinforced Shoe Midsoles with Balanced Stiffness/Damping Behavior

**DOI:** 10.3390/ma17020401

**Published:** 2024-01-13

**Authors:** Michael Cordin, Sandra Eberle, Thomas Bechtold, Christian Bitschnau, Kevin Lins, Fabien Duc, Raphaële Chapuis, Tung Pham

**Affiliations:** 1 Research Institute of Textile Chemistry and Textile Physics, University of Innsbruck, Hoechsterstrasse 73, 6850 Dornbirn, Austria; 2 INNOcomposites GmbH, Klarenbrunnstraße 115, 6700 Bludenz, Austria; 3 POMOCA SA, Route de Préverenges 14, 1026 Denges, Switzerland

**Keywords:** shoe sole damping, loss modulus, storage modulus, viscoelastic, hemp fibers

## Abstract

The comfort of walking depends heavily on the shoes used. Consequently, the midsole of shoes is designed in such a way that it can dampen force peaks during walking. This significantly increases the overall wellness during walking. Therefore, the midsole usually consists of rubber-like polymers, such as polyurethane and ethylene-vinyl acetate copolymer. Furthermore, the manufacturing process of these polymers results in a foam-like structure. This further enhances the damping behavior of the material. Nevertheless, it would be desirable to find a cheap and sustainable method to enhance the damping behavior of the shoe midsole. The purpose of this work is to see if hemp fibers, which are part of the polymer matrix material, could improve the stiffness without losing the damping behavior. The mechanical properties of such prepared fiber-reinforced composites were characterized by quasi-static tensile testing and dynamic mechanical analysis. The mechanical properties were examined in relation to the fiber type, weight fraction, and type of polyurethane used. Furthermore, the investigation of the embedding of these fibers in the polymer matrix was conducted through the utilization of optical and electron microscopy.

## 1. Introduction

The comfort of walking depends heavily on the material design of the shoe soles. Usually, the upper, midsole, and outsole are the three main components of a shoe. Each component has its own function, so the outsole is responsible for good contact between the shoe and the ground, and the midsole damps force peaks during walking [1]. Therefore, the midsole fulfills a crucial function: it enables good overall wellness during walking. Especially when walking on stiff ground, shoes with good damping properties are highly recommended [2].

Usually, the midsole is made of polymeric foamed elastomeric material; the standard polymers are polyurethane and ethylene-vinyl acetate (EVA) copolymers [1,3]. Thus, it is important to characterize the mechanical properties of these materials, which enables the possibility of designing shoes with improved damping behavior. In the selection of a suitable material for the midsole, it is also important to consider the aging resistance of the material because, during the lifetime of the shoe, many loading and unloading can occur [4]. Usually, with the increasing aging of the shoe, the stiffness of the midsole increases, and its maximum deformability and energy loss during walking decrease [4,5,6]. This material fatigue is explained by the folding and breakout of the cell walls in the polymeric foam material [7].

**Figure 1:**
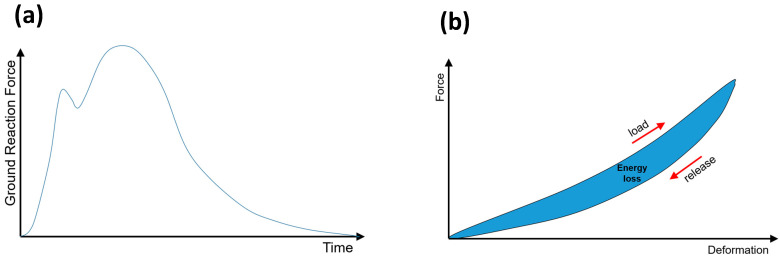
Ground reaction force as a function of time during a running step and the resulting contact of the shoe sole with the ground (**a**) and hysteresis curve in the force-deformation diagram (**b**). The energy loss corresponds to the enclosed area of the hysteresis curve.

During a running step, the force introduced into the ground varies with time (see Figure 1a) [8]. A first force peak occurs during the contact of the back-food area with the ground. This force consists of the body’s weight and the necessary force to decrease the speed of the moving body part towards the ground. For a running speed of 3.5 m/s, this force can correspond to the double body weight [6].

The ground reaction force during a running step leads to a deformation of the shoe midsole. Consequently, energy is stored in the elastic material. During the lifting of the food and unloading of the shoe material, energy is released due to the expansion of the previous compressed midsole material (see Figure 1b). The enclosed area of the hysteresis curve of the loading and unloading sequence corresponds to the energy loss during a running step. This loss of elastic potential energy can be explained by the production of heat.

But it also seems possible that a part of the stored energy in the shoe is returned to the walker, and through a spring-like repulsion, the velocity of the walker is increased; this would result in more energy-efficient walking and running. With this end in mind, to save energy, special shoes were already developed [9]. These shoes lead to improved running times in marathon runs, but it is still controversial if this is caused by a return of stored energy in the shoe to the runner [10,11]. An energy-saving walk is only possible if the stored energy in the shoe is returned to the walker in a suitable direction, time, and frequency [12]. However, it was estimated that the amount of stored and returned energy in the muscle-tendon system of the legs is, in any case, much larger than that in typical shoes [13]. However, a good damping of the shoe can maybe reduce the muscular work to stabilize the walking and, therefore, increase the general well-being [14,15]. So it was shown that shoes with air-sole material instead of standard EVA midsoles lead to decreased oxygen consumption by the runner [16].

Consequently, it seems desirable to design shoes in such a way that their damping properties are improved.

It is evident that high shoe weight increases the physical exertion of an athlete during a short run at high speed [17,18,19,20]. Therefore, for sports applications, it is also important to design the shoe with a lightweight material.

The present work aims to improve the damping behavior of polyurethane matrixes by embedding hemp fibers. Such prepared composites, reinforced with hemp fibers, are already described in the scientific literature, but to the knowledge of the authors, there are no detailed reports about the damping properties of such composites. Due to environmental reasons, the number of reports about hemp and flax fiber-reinforced composites has increased during the last few years. Such fibers were for example used to reinforce epoxy [21,22,23,24,25,26,27,28,29,30,31,32,33,34,35], polyester [36,37,38,39,40,41,42,43,44], polyethylene [45,46,47,48,49,50], polypropylene [51,52,53,54,55,56,57,58], polyurethane [59,60,61], and polyamide [62,63] matrixes.

## 2. Materials and Methods

### 2.1. Materials and Sample Preparation

In the present work, three different two-component-polyurethane systems with different shore hardness are supplied from Sika Deutschland GmbH (Bad Urach, Germany) and were used: SikaBiresin U1404 (Shore A 40) based on isocyanate/amine combination, SikaBiresin UR340 (Shore A 60) based on isocyanate/polyol combination and SikaBiresin UR3450 (Shore A 80) based on isocyanate/polyol combination.

The natural fibers used in the study are retted and cardered hemp long fibers. Hemp fibers were provided by Seilerei Eisserer (Amstetten, Austria) with a mean fiber bundle diameter of around 136 µm. The fibers were cut into short fibers with a length of 20 mm and randomly mixed in the polyurethane formulations in order to minimize the anisotropy of the composites. The fiber weight fraction was varied up to 10%. The polymer/fiber composite sheets with a thickness of 5 mm were produced by mold casting at 25 °C. The sheets were molded after 2 h, followed by further curing up to 6 days.

### 2.2. Analytical Methods

Quasi-static tensile tests were conducted using the Zwick-Roell Z010 device (Ulm, Germany), which is suitable for breaking samples up to a force of 10 kN. The test standard used was DIN EN ISO 527-4 [64]. The sample dimensions were 80 × 10 × 5 mm and the clamping length was 20 mm during the measurement. In the beginning, the tensile tests were performed at a speed of 5 mm/min to determine the elastic modulus and afterward, the speed was increased to determine the elongation at UTS. These measurements determined the following parameters: elongation, elastic modulus, and ultimate tensile strength (UTS). Experimentally determined stress-strain curves were used to calculate all these physical parameters.

The values presented are the average of three samples, and the standard deviations were calculated, which are depicted as error bars in the corresponding figures.

The different composites were characterized by dynamic mechanical analysis (DMA) with the Anton-Paar MCR302 rheometer (Graz, Austria). Storage and loss moduli were determined in a frequency sweep measurement with a shear strain of 0.02%. The sample width and thickness were 10 and 5 mm, respectively, and the clamping length was 30 mm.

Optical micrographs of prepared composites were taken using an Olympus CX-41 microscope (Tokyo, Japan). An Olympus XC50 digital camera (Tokyo, Japan) was used to capture the images. The cross-section was additionally characterized by the SEM images, which were captured by the Hitachi TM4000Plus electron microscope (Tokyo, Japan).

## 3. Results and Discussion

For fiber-reinforced composites, a high value for the ratio of fiber surface to fiber volume is desirable because, due to the high contact area between fiber and the surrounding matrix, the matrix can efficiently transfer the stress to the fiber, leading to a failure of the fiber and not an undesired pull-out of the fiber by applying a large force to the composite. This ensures that the full reinforcing potential of the fibers can be realized.

A high value for the elastic modulus and tensile strength is not the most important thing for fiber-reinforced shoe midsole composites. Good damping properties are more important. In the present work, it was investigated if the damping properties of a midsole of a given polymer can be improved by the embedding of fibers in the polymer matrix. Due to sustainable reasons, the potential of natural fibers, namely hemp, was investigated to improve the stiffness and damping properties of polyurethane. This strategy seems to be a cost-effective way to improve these properties of polyurethane.

**Figure 2:**
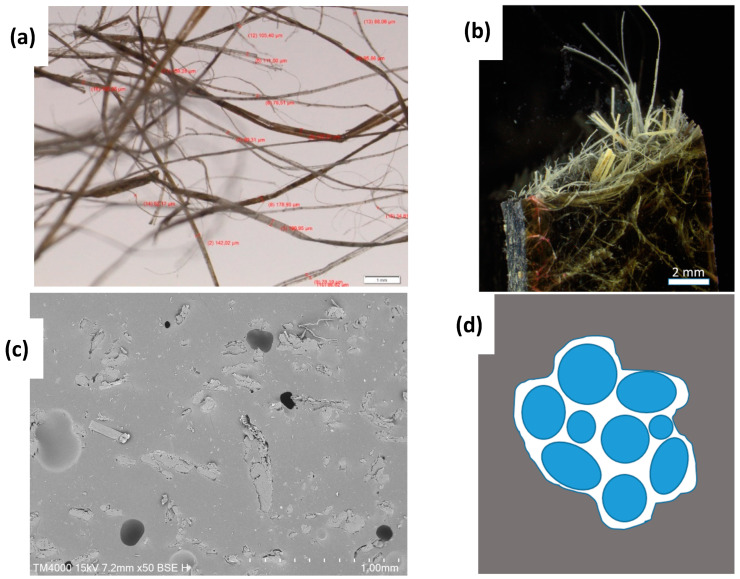
Optical microscopies of hemp fiber (**a**), broken surface of hemp reinforced composite (**b**), SEM image of the cross-section of embedded hemp fiber bundles in a polyurethane matrix (**c**), and schematic representation of a fiber bundle in polymer matrix material (**d**).

Hemp fibers are renewable plant fibers. These fibers owe their strength to cellulose molecules, and they are often used in textile applications in the form of fiber bundles. Fibre bundles are aggregates of several single fibers stuck together by pectin and lignin (Figure 2a). Figure 2b shows a broken composite reinforced with hemp fibers. The outstanding strings consist of fiber bundles. Figure 2c shows the cross-section of embedded flax fibers in a polyurethane matrix. It can be easily recognized that the embedded aggregates consist of several fibers, and therefore, they consist of fiber bundles. Therefore, the SEM image of Figure 2c confirms that the fiber bundles are still intact and that their fibers are not separated by the penetration of resin during the composite preparation.

The use of fiber bundles instead of single fibers has advantages for improving the damping properties of polyurethane. The embedding of single hemp or flax fibers in a polyurethane matrix will increase the stiffness, but an increased stiffness is not automatically connected to a good damping behavior of the material. Figure 2d shows a sketch of the cross-section of a fiber bundle, embedded in a polymer matrix. The fibers in the fiber bundle are in close contact, stuck together partially by pectin and lignin. Therefore, it can be assumed that the matrix components cannot fill out the space between the fibers during the composite preparation.

Consequently, the adhesion between the fibers in the fiber bundle is not strong, and it can be assumed that the fibers can glide along each other if the material is compressed or expanded. This movement relates to friction and causes energy dissipation in the form of heat, and this material is less stiff than single-fiber reinforced polymers. This energy dissipation enhances the damping properties of the material, so the use of fiber bundles instead of single fibers seems advantageous for this purpose. Besides, the processing of hemp plants into fiber bundles instead of single fibers is easier from a technical point of view and is cheaper from an economic point of view.

During the composite preparation, the interaction between the fibers and the liquid resin underlies a competition between surface energy and elastic strain energy. The resulting capillary adhesion can influence the arrangement of the reinforcing fibers; a theoretical/experimental analysis of this process can be found in the literature [65].

The mechanical characterization of the different composites with tensile testing is important to judge their robustness under strong loading, which can be important for sports applications and special work shoes.

The embedding of fibers in the form of fiber bundles alters the mechanical properties of polyurethanes, and this was characterized by quasi-static tensile testing. It must be noted that, typically, the mechanical properties of natural fibers vary much more strongly compared to synthetic fibers. Besides, the number of single fibers in the used fiber bundles also varies. Therefore, the determined mechanical properties are scattered accordingly around the mean value.

**Figure 3:**
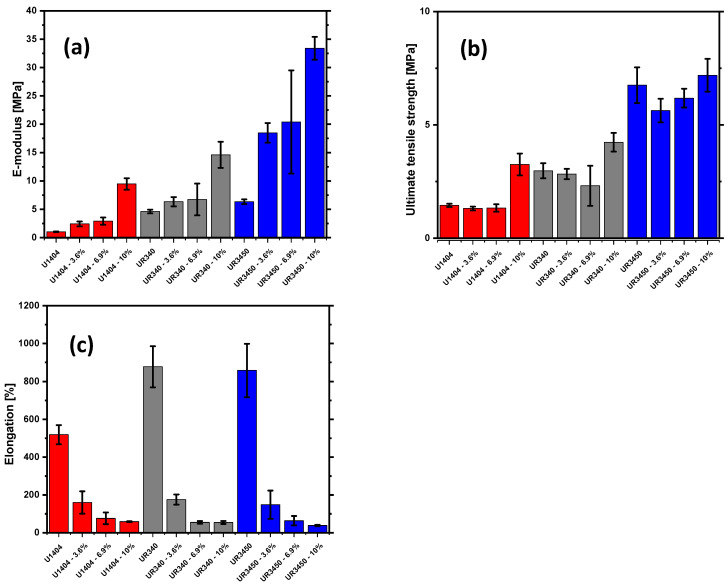
Elastic moduli (**a**), ultimate tensile strength (**b**), and elongation (**c**) of the different composites with different types of polyurethane and different amounts of reinforcing hemp fibers.

Twelve different hemp-fiber-reinforced composites were prepared with three different types of polyurethane. The used fiber length was between 10 and 20 mm. The three types of polyurethane are referred to as the U1404, UR340, and UR3450, and each of these different polymer matrixes is reinforced with a fiber content of 0%, 3.6%, 6.9%, and 10%, respectively. Figure 3a shows the determined elastic modulus values for the different composites. It can be seen from the data that the elastic modulus of pure polymers increases according to the following series: U1404 < UR340 < UR3450.

Additionally, it can be seen from Figure 3a that the elastic modulus increases strongly with an increasing amount of added hemp fibers. The stiffness of hemp fibers is higher than that of pure matrix material, and therefore, this trend is expected. It should therefore be considered that the embedding of hemp fibers into the shoe sole matrix polymer increases the stiffness, and consequently, the shoe sole becomes less soft. Nevertheless, the elastic modulus values are comparatively minimal for all investigated materials, so all materials are soft.

It could be observed that the ultimate tensile strength (UTS) increases for pure polymer matrix according to the following series: U1404 < UR340 < UR3450 (see Figure 3b). Embedded hemp fibers have a limited effect on the UTS of these composites, in contrast to the elastic modulus. An increased UTS would be desirable for the shoe midsole because this would increase the life expectancy, especially with increased loading, which can, for example, occur for hiking shoes. Nevertheless, the addition of these fibers does not worsen the UTS values, and therefore, such modified materials are suitable to produce shoe midsoles from this point of view.

Figure 3c shows the elongation at UTS values of the different composites. The pure polymer materials are soft and can be stretched strongly until they break. The elongation at break of these three materials lies between 400 and 900%. The addition of hemp fibers decreases the elongation strongly, and with increasing fiber content, the elongation decreases correspondingly.

The present work aims to improve the stiffness without significantly worsening the damping properties of polyurethane by embedding hemp fibers. This seems to be desirable to protect the environment, to spare resources, and for cost-efficient production. The effect of these natural fibers on damping properties can be characterized by dynamic mechanical analysis (DMA). In these measurements, the storage modulus and loss modulus were determined.

The measured force to determine the storage modulus acts versus a spring-like mechanism, and therefore, the corresponding needed energy is stored in this spring-like structure and can be recovered. This is the reason for the term “storage modulus”. In contrast, the measured force to determine the loss modulus acts against a damper-like mechanism, and consequently, the corresponding needed energy is lost due to energy dissipation. Therefore, the larger the amount of energy dissipated by a material, the larger the ratio of loss modulus to storage modulus.

**Figure 4:**
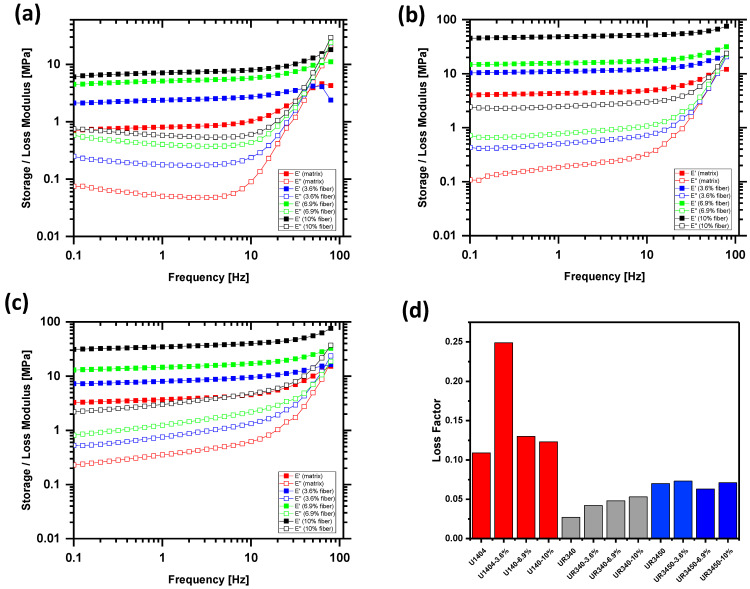
Storage modulus *E*′ and Loss modulus *E*″ for composites with different amounts of hemp fibers embedded in U1404 matrix (**a**) UR340 matrix (**b**) and UR3450 matrix (**c**). Red: matrix only: blue: 3.6% fiber; green: 6.9% fiber and black:10% fiber. Solid symbols: storage modulus *E*′: open symbols: loss modulus E″. Figure (**d**) shows the loss factor at 0.1 Hz for the different materials; the loss factor for the U1404-3.6%-composite seems to be an outlier, probably due to fiber distribution inhomogeneity.

Figure 4 shows the storage modulus *E*′ and loss modulus *E*″ for composites of polyurethane matrix reinforced with hemp fibers. *E*′ and *E*″ are plotted versus the frequency, which characterizes the periodic loading of the material. The results indicate that the storage modulus as well as the loss modulus increase with an increasing amount of embedded hemp fibers. The increase in the storage modulus indicates that the material becomes stiffer with an increasing amount of hemp fibers, and therefore, these results confirm the findings from the quasi-static tensile testing. Besides, the results indicate that the storage and loss modulus increase with increasing frequency. This relation between storage/loss modulus and frequency is important to characterize the physics of the shoe sole material as a function of walking speed. With increasing walking speed, the frequency of loading and unloading of the shoe sole material increases. The loss modulus (and consequently the ability of the material for energy dissipation) increases with increasing frequency, and this is desirable because, with increasing walking speed, the force peaks increase during stepping on the ground.

Although the increased stiffness of the material is due to the addition of fibers, damping is still clearly present because of the increased loss modulus. The ratio of loss modulus to storage modulus is called the loss factor and describes the ratio of the introduced energy, which is supplied to the spring-like and damper-like structures, respectively. The higher the ratio, the higher the proportion of energy lost by energy dissipation in the damper-like structure. The higher the proportion of energy lost by energy dissipation, the better the damping of the material. Therefore, the addition of hemp fibers has the potential to strongly enhance the stiffness of the material without losing its damping properties. Shoes with such a reinforced midsole can, for example, be used for hiking shoes, where a stiff but also damping sole is desirable.

These assumptions are based on the viscoelastic model. The stress change can be described by a Taylor series of the strain *ε* and a Taylor series of *dε*/*dt*. Consequently, only for small stress values, there is a linear relation between the stress and *ε* and *dε*/*dt*. Therefore, the composites were first characterized with an amplitude sweep measurement to find out the stress range, where the viscoelastic model can be used to estimate the material’s properties (see the later calculations).

The values of the loss factor for the different prepared materials can be seen in Table 1. In principle, the higher the loss factor, the higher the lost energy due to energy dissipation is, and consequently, the better the damping property. Although the stiffness of the material is increased strongly by the addition of many fibers, the loss factor remains more or less unchanged or is even a little bit increased. This indicates that the addition of hemp fibers in the form of fiber bundles is a good strategy to increase the stiffness of shoe midsole material without losing its damping properties.

A part of the energy input into the shoe midsole is stored, and another part is dissipated in the form of heat. Therefore, the mechanics of the shoe midsole can be described by a viscoelastic model [13]. Energy is stored in elastically deformed materials, and heat is produced by overcoming the viscous resistance. The effect of these two processes can be modeled by a purely elastic spring and a purely viscous damper connected in parallel and is known as the Kelvin–Voigt model [66]. But it must be kept in mind that not all stored energy during walking is stored in the midsole compression because a part of the energy is stored in the bending of the shoe.

The resistance of a viscoelastic material against deformation is due to its spring-like but also damper-like behavior.

The Kelvin-Voigt model assumes that the spring and damper are connected in parallel, and therefore, an applied force causes the same strain on the two components. The total stress $\sigma_{Total}$ is:

(1) $$\sigma_{Total}=\sigma_{spring}+\sigma_{damper}$$

 σ(t) and $\varepsilon \left(t\right)$ fulfill the following equation:

(2) $$\sigma \left(t\right)=E\epsilon \left(t\right)+\eta \frac{d\epsilon (t)}{dt}.$$

*E* is the Young’s modulus and *η* is the viscosity. The term $\eta \frac{d\varepsilon }{dt}$ describes the stress caused by energy dissipation by overcoming the viscous resistance, meanwhile, the term Eϵ describes the “reversible” part of the stress. Therefore, a cyclic movement with compression and expansion of the material leads to a hysteresis in the stress-strain diagram, and consequently, the energy loss can be explained by the viscous resistance.

If the stress $\sigma_{0}$ (at the time *t* = 0) is applied to a material, then it is possible to determine the strain ϵ as a function of time:

(3) $$\epsilon \left(t\right)=\frac{\sigma_{0}}{E}\left(1-e^{-t/\tau }\right).$$

With $\tau =\frac{\eta }{E}$. So, at the time *t* = 0, the strain ϵ=0, but with increasing time, the strain goes versus $\frac{\sigma_{0}}{E}$, and reaches, therefore, the same value as a purely elastic material. But due to viscous resistance, less mechanical energy can be gained from the movement from ϵ=0 to $\epsilon =\frac{\sigma_{0}}{E}$.

In analogy to Formula (2), the stress *σ* during the dynamic mechanical analysis can be estimated as a function of the shear strain ɛ and its time derivative with the following formula (note that the time derivative of $\epsilon_{0}\sin\omega t$ is $\varepsilon_{0}\omega \cos\omega t$):

(4) $$\sigma \left(t\right)=\epsilon_{0}\left(E^{\prime }\sin\omega t+E^{\prime \prime }\cos\omega t\right).$$

*E*′ and *E*″ are the storage and loss modulus, respectively. *ω* is the angular frequency and ɛ_0_ is the maximum shear strain during the rheometer measurement. Note that the shear strain in the rheometer measurement corresponds to a twisting of the material. The shear strain *ɛ*(*t*) during the rheometer measurement fulfills the following equation:

(5) $$\;\;\varepsilon \left(t\right)=\epsilon_{0}\sin\omega t.$$

**Figure 5:**
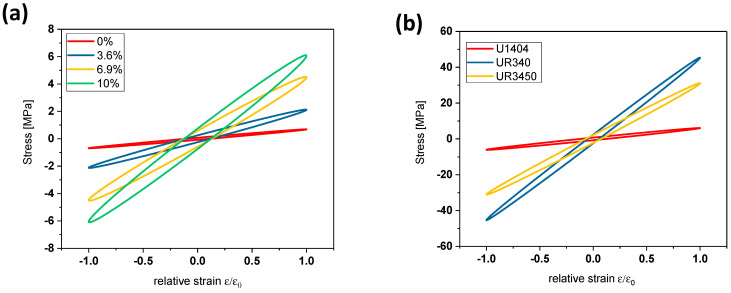
Hysteresis of the stress-strain relation for U1404 composites as a function of the hemp fiber content (**a**) and comparison between 10% hemp composites with different matrixes (**b**). Note: negative values of stress correspond to a twisting of the material in the opposite direction.

From the determined storage and loss modulus (at the frequency of 0.1 Hz) and with the help of Formulas (4) and (5), it is possible to determine the hysteresis of the stress-strain relation. Figure 5a shows the hysteresis of the hemp fiber-reinforced U1404-composites as a function of fiber content. The enclosed area of the hysteresis is proportional to the amount of dissipated energy, and it can be seen clearly from Figure 5a that this amount of energy increases strongly with increasing amounts of hemp fibers.

The damping increases with increasing fiber content. Figure 5b shows the hysteresis of composites with a fiber content of 10% but with different polyurethane matrixes. It can be observed that the highest damping can be reached with the UR340 polyurethane matrix.

## 4. Conclusions

For special applications, it can be desirable to design shoes with a stiff shoe midsole. Polyurethane is a common polymer used in the fabrication of shoe midsoles. The present work shows that the embedding of natural fibers, in the form of hemp fibers, can make the polymer matrix stiffer while maintaining good damping properties. The use of natural fibers is a sustainable and cheap method to achieve this goal and can help save synthetic polymers to produce shoe soles. It is assumed that the use of natural fibers in the form of fiber bundles enhances the damping properties due to increased friction between the fibers. The use of fiber bundles instead of single fibers is resource-saving and promises even better properties. To find out which composite is best suited as a shoe sole material, a characterization with quasi-static tensile testing and dynamical mechanical analysis (DMA) is useful. These analyses show the elastic modulus of polyurethane can be increased strongly by the embedding of hemp fibers, but the characterization with DMA shows that this increased stiffness is not combined with a decrease in damping behavior. This property is desirable, especially, for example, for hiking and work shoes.

## Figures and Tables

**Table 1:** Loss factor at 1 Hz for the different composites with different matrix materials and reinforced with different amounts of hemp fibers.

Base Material	Fiber Content (%)	Loss Factor
U1404	0	0.063
	3.6	0.075
	6.9	0.078
	10	0.081
UR340	0	0.043
	3.6	0.046
	6.9	0.049
	10	0.051
UR3450	0	0.096
	3.6	0.094
	6.9	0.086
	10	0.087

## Data Availability

Data are contained within the article.
